# KDP, a Lactobacilli Product from Kimchi, Enhances Mucosal Immunity by Increasing Secretory IgA in Mice and Exhibits Antimicrobial Activity

**DOI:** 10.3390/nu13113936

**Published:** 2021-11-04

**Authors:** Mamdooh Ghoneum, Shaymaa Abdulmalek

**Affiliations:** 1 Department of Surgery, Charles R. Drew University of Medicine and Science, Los Angeles, CA 90059, USA; 2 Department of Biochemistry, Faculty of Science, Alexandria University, Alexandria 21511, Egypt; shimaa_salamy@yahoo.com

**Keywords:** IgA, secretory, KDP, Peyer’s patches, OVA albumin, antioxidant, antimicrobial

## Abstract

The potential of KDP, a lactic acid bacterial strain of *Lactobacillus sakei*, to enhance the production of mucosal specific immunoglobulin A (IgA) in mice and thereby enhance gut mucosal immunity was examined. KDP is composed of dead cells isolated from the Korean traditional food kimchi. Female BALB/c mice orally received 0.25 mg KDP once daily for 5 weeks and were co-administrated ovalbumin (OVA) for negative control and cholera toxin for positive control. Mice administered KDP exhibited increased secretory IgA (sIgA) contents in the small intestine, Peyer’s patches, serum, colon, and lungs as examined by ELISA. KDP also significantly increased the gene expression of *Bcl-6*, *IL-10*, *IL-12p40*, *IL-21*, and *STAT4*. In addition, KDP acted as a potent antioxidant, as indicated by its significant inhibitory effects in the range of 16.5–59.4% for DPPH, nitric oxide, maximum total antioxidant capacity, and maximum reducing power. Finally, KDP exhibited potent antimicrobial activity as evidenced by a significant decrease in the growth of 7 samples of gram-negative and gram-positive bacteria and *Candida albicans*. KDP’s adjuvant effect is shown to be comparable to that of cholera toxin. We conclude that KDP can significantly enhance the intestine’s secretory immunity to OVA, as well as act as a potent antioxidant and antimicrobial agent. These results suggest that orally administered KDP should be studied in clinical trials for antigen-specific IgA production.

## 1. Introduction

Many attempts have been made to induce mucosal immunity in order to enhance its protection against invasion by pathogens and to neutralize toxins at mucosal surfaces [1]. As part of a host’s acquired immune defense to infections, harmful bacteria and viruses can be neutralized by mucosal immunoglobulin A (IgA). This antibody plays a key role by interfering with bacterial and viral motility and with their attachment to epithelial cells [2]. IgA-committed B cells can be initiated and generated by Peyer’s patches, lymphoid tissues associated with the gut [3]—in Peyer’s patches, follicular helper T (Tfh) cells regulate the development of IgA+ B cells and the production of antigen-specific IgA [1]. These Tfh cells are in turn affected by a variety of molecular expression levels, including those of *STAT3*, cytokines *IL-6* and *IL-21*, and surface markers PD-1 (programmed death 1), ICOS (inducible co-stimulator), and CXCR5 (CXC chemokine receptor 5) [4,5].

Recent studies have investigated the improvement of host defense in the saliva and gut due to modifications of IgA production by products such as probiotics, fermented foods, synbiotics, parabiotics, and other microbial forms [6,7]. Probiotics are generally lactic acid bacteria (LAB). Our work and that of others have shown that LAB can provide several health benefits. Several LAB strains can modulate the immune system by increasing immunoglobulins and exerting pro- and anti-inflammatory effects [8]. In addition, LAB strains can inhibit the growth of pathogenic bacteria [9,10,11], reduce viral infectivity [12,13], or even act as anticancer agents [14], and reviews have shown that LAB strains have the potential to serve as alternative or complementary treatment options for infection and inflammatory diseases [15].

The current study was carried out to examine if KDP, a LAB strain of *Lactobacillus sakei*, can enhance IgA production in mice. KDP is composed of dead cells isolated from the Korean traditional food kimchi. The effects of orally administered KDP on mice were examined by measuring IgA contents as well as the gene expression levels of *Bcl-6*, *IL-10*, *IL-12p40*, *IL-21*, and *STAT4* associated with Tfh cell differentiation. In addition, the antioxidant and antimicrobial activities of KDP were also examined.

## 2. Materials and Methods

### 2.1. Mice

Forty female BALB/c mice were used. Mice were 25–30 g in weight and 5 weeks old. They were obtained from the National Research Center’s animal facility in Cairo, Egypt. Mice were housed 6 per cage and allowed to acclimate for one week before the study’s start. Their environment was carefully controlled at constant temperature (25 ± 2 °C), relative humidity (60 ± 10%), and light/dark cycles (12/12 h). The discomfort and suffering experienced by mice were kept to a minimum.

### 2.2. Lactic Acid Bacteria KDP

KDP is composed of dead cells from the bacterial strain *Lactobacillus sakei*, which are isolated from the Korean traditional food kimchi. After a fermentation process, the resulting culture is centrifuged, removed, and washed out, and the bacteria are killed with heat. KDP is then prepared as a freeze-dried powder. Daiwa Pharmaceutical Co. Ltd. (Tokyo, Japan) kindly provided the KDP. This powder contained 1 × 10^12^ bacteria/g. The powder was diluted 100 times with malt dextrin until 1 × 10^10^ bacteria/g which was used in this study. KDP was prepared in saline (0.9% *w*/*v*) and the solutions were freshly prepared each day.

### 2.3. Chemicals

The following chemical products were used in our study: total Mouse IgA ELISA kit (Cat# MBS564073), OVA-specific IgA ELISA kit (Cat# MBS2600003), QIAzol lysis reagent (Cat# 79306), RNeasy Mini Kit (Cat# 74104) (QIAGEN, Hilden, Germany), SensiFAST SYBR One-Step Kit (BIO-72001) (Meridian Bioscience, Cincinnati, OH, USA), Primers (Sigma-Aldrich, St. Louis, MO, USA), Ovalbumin (OVA; gradeV, Sigma-Aldrich, St. Louis, MO, USA), Freund’s Complete Adjuvant, Cholera toxin, Protease inhibitor cocktail, Trypsin inhibitor, and HEPES (Sigma-Aldrich, St. Louis, MO, USA).

### 2.4. Experimental Design

**Figure 1:**
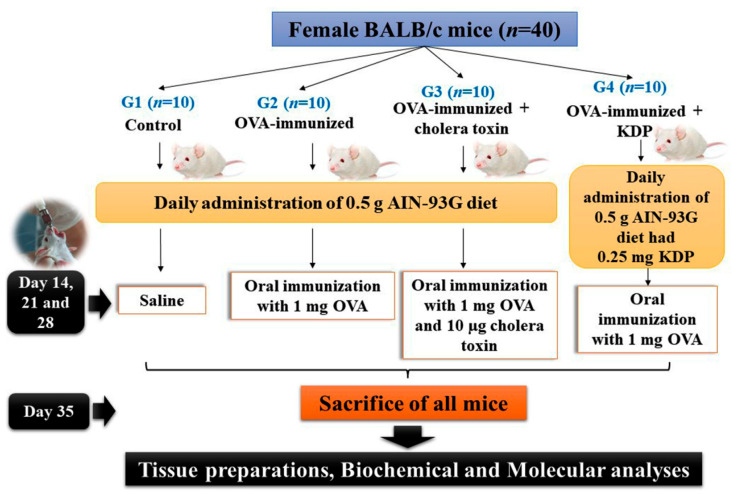
Schematic diagram of experimental design.

Mice were divided into 4 groups with 10 mice per group (Figure 1). Group 1 (Control) served as the negative control; these mice were fed a 0.5 g AIN-93G diet independently from their usual diet for 5 weeks. Group 2 (OVA) was the ovalbumin (OVA)-immunized group; these mice were fed a 0.5 g AIN-93G diet once per day for 5 weeks and orally immunized by means of intragastric gavage with 1 mg of OVA on days 14, 21, and 28. Group 3 (OVA-CT) was the OVA-immunized + cholera toxin group and served as the positive control group; these mice were fed a 0.5 g AIN-93G diet once per day for 5 weeks and orally immunized with 1 mg of OVA and 10 μg of cholera toxin on days 14, 21, and 28. Group 4 (OVA-KDP) was the OVA-immunized + KDP group; these mice were orally administered a 0.5 g AIN-93G diet that had 0.25 mg KDP once daily for 5 weeks, and orally immunized with 1 mg OVA on days 14, 21, and 28. We estimated that approximately all 0.5 g AIN-93G diet were given orally to each mouse daily and consumed completely because we added it before normal pellet diet and throughout the feeding period, mice ingested about 2 g of diet per day. Mice in all groups were sacrificed on day 35 and sera, small intestines, intestinal lavage, Peyer’s patches, colons, colon contents, and lungs were collected from all mice.

### 2.5. Preparation of Tissues

Tissues from the small intestine and colon were longitudinally opened, and all contents were removed and washed with PBS. The small intestine, standard area of the proximal part of descending colon, and lung tissues were homogenized in PBS (1 mL) containing a protease inhibitor cocktail, 40 mM HEPES, 1% Triton, and 10% glycerol, then centrifuged at 4 °C for 15 min at 12,000 rpm, and the supernatant was then collected. Peyer’s patches were isolated from the small intestines. Lavage fluid from the small intestines was collected by washing out the intestines with PBS, followed by centrifugation of the fluid at 4 °C for 15 min at 12,000 rpm and subsequent collection of the supernatant. Then the colon contents from each mouse were collected and suspended in a specific buffer (PBS containing 0.1 mg/mL of a trypsin inhibitor and 50 mM EDTA), vortexed vigorously, and centrifuged again at 13,000 rpm for 15 min, and the supernatant was collected. A mouse IgA ELISA was used to detect total IgA and OVA-specific IgA in all collected supernatants. Supernatant protein levels were quantified via the BCA Protein assay kit using BSA as a standard.

### 2.6. Cytokine and IgA Measurements

#### 2.6.1. Determination of Total IgA

Examination of total IgA was conducted in accordance with the manufacturer’s protocol for the commercial kit. The Standard was gently stirred for 15 min before using it to craft serial dilutions (320, 160, 80, 40, and 20). Briefly, 100 μL of Standard, Blank, or Sample (in triplicate) was added to each well, enclosed with the plate sealer, and incubated for 60 min at room temperature. The plate was aspirated and washed with diluted wash solution 5 times. Then the liquid was separated from the wells and 100 μL of the diluted enzyme-antibody conjugate was added to each well and incubated at room temperature for 30 min. The plate was again aspirated and washed with diluted wash solution 5 times. The plate was then incubated for 10 min at 37 °C in the dark after the addition of 100 μL TMP substrate solution to each well. The reaction was stopped with 50 μL of Stop Solution, and the absorbance in a microplate reader was measured at 450 nm. A standard curve was constructed from which the concentration of total IgA was then determined as pg/mg protein.

#### 2.6.2. Determination of OVA-IgA

Examination of OVA-IgA was conducted in accordance with the manufacturer’s protocol for the commercial Kit. The Standard was gently stirred for 30 min before using it to craft serial dilutions (100, 50, 25, 12.5, 6.25, 3.12, and 1.56 U/mL). Briefly, 100 μL of Standard or Sample (in triplicate) was added to each well, enclosed with adhesive tape, and incubated for 90 min at 37 °C. The plate was aspirated and washed two times. Then 100 μL of biotinylated Mouse OVA IgA antibody liquid was added to each well, sealed with adhesive tape, and incubated at 37 °C for 60 min. The plate was aspirated and washed 5 times. After that, 100 μL of color reagent solution was added to each well (including blank well), the plate was incubated for not more than 30 min at 37 °C in the dark. Then 100 μL of color reagent C was added to each well (including blank well), the plate was mixed, and the absorbance was measured at 450 nm within 10 min. A standard curve was constructed from which the concentration of OVA-IgA was then determined as U/mg protein.

#### 2.6.3. RNA Extraction and RT-PCR Analysis

Total RNA was extracted from Peyer’s patches and small intestine samples using QIAzol lysis reagent as directed by the manufacturer and then isolated using the RNeasy Mini Kit. SensiFAST™ SYBR (Meridian Bioscience, Cincinnati, OH, USA) was used to accomplish quantitative real-time RT-PCR. The cDNA synthesis reaction was carried out at 45 °C for 10 min, following which the polymerase activation was performed at 95 °C for 2 min. After adjusting all the parameters, the primers were designed using the primer designing tool NCBI Primer-BLAST software (National Center for Biotechnology Information, Bethesda, Rockville, MA, USA). The primers (Table 1) were then subjected to PCR cycles, denaturation at 95 °C for 5 s, and annealing/extension at 60 °C for 20 s.

### 2.7. Antioxidant Activity

KDP’s antioxidant activity was measured using four different assays: DPPH, nitric oxide, reducing power assay, and total antioxidant capacity (TAC).

#### 2.7.1. Determination of DPPH Scavenging Activity

The ability of KDP to scavenge DPPH radicals was assessed by using the method of Blois [16]. The stock solution of 0.2 nmoL/DPPH was prepared in methanol and kept at −20 °C until analysis. Briefly, 0.5 mL of prepared solution was added to different concentrations of KDP (5–100 µg/mL) and standard (ascorbic acid). The reaction mixture was vortexed completely and kept for 30 min in the dark at room temperature. Then the reaction mixture absorbance was measured at 517 nm. The ability of KDP to scavenge DPPH radical was calculated by the equation: DPPH radical scavenging activity:

% scavenging =(A0− A)/A0×100

where A0 is the absorbance of the control and A is the absorbance of the test sample.

#### 2.7.2. Determination of NO Radical Scavenging Activity

The Griess Illosvoy reaction was used to measure nitric oxide radical inhibition [17]. The Griess reaction was used to detect nitric oxide generated from sodium nitroprusside. Sodium nitroprusside spontaneously produces nitric oxide in aqueous solution at physiological pH, which reacts with oxygen to produce nitric ions that may be measured with the Griess reagent. To summarize, 400 μL sodium nitroprusside (10 mmol/L) and 100 μL phosphate buffer 0.2 mmol/L, pH 7.4, were added to different concentrations of KDP and standard. In a dark chamber, this reaction mixture was incubated for 2.5 h at 25 °C. After that, 500 μL of Griess reagent (1% sulphanilamide, 2% orthophosphoric acid, 0.1% N-1-naphthylethylenediamine dihydrochloride) was added. The absorbance was measured at 546 nm, and the scavenging activity percentage was computed as described before.

#### 2.7.3. Reducing Power Assay

The reducing power of KDP was determined by the method described previously [18]. Briefly, 2.5 mL phosphate buffer (0.2 M) and 2.5 mL potassium ferricyanide (1%) were mixed with various amounts of KDP and ascorbic acid. At 50 °C, the KDP and ascorbic acid were incubated for 20 min. After cooling, 2.5 mL trichloroacetic acid (10%) was added to the mixtures, which were then centrifuged at 3000 rpm for 10 min. Finally, 2.5 mL of the supernatant was combined with 0.5 mL of freshly produced FeCl3 (0.1%). The reaction mixtures’ absorption was then measured at 700 nm. As with the DPPH assay, the reducing power assay percentage was computed.

#### 2.7.4. Total Antioxidant Capacity

The total antioxidant activity of KDP was determined using the phosphomolybdate test method [19]. One hundred microliters of KDP was added to a reagent solution containing sulphuric acid (0.6 M), sodium phosphate (28 mM), and ammonium molybdate (4 mM) and incubated at 95 °C in a water bath for 90 min. The absorbance was measured at 765 nm against a reagent blank after cooling. As a reference, we calculated the total antioxidant capacity of ascorbic acid. The inhibition was estimated according to the instructions in the DPPH assay.

### 2.8. Antimicrobial Activity

The antibacterial activity of KDP was determined using an agar well diffusion assay [20] with three gram-negative bacteria (*Klebsiella pneumonia* ATCC700603, *Salmonella typhi* ATCC14028, and *Escherichia coli* ATCC25922), three gram-positive bacteria (*Staphylococcus aureus* ATCC25923, *Streptococcus pyogenes* EMCC1772, and *Bacillus cereus* ATCC10876), and one yeast strain (*Klebsiella pneumonia* ATCC700603, *Salmonella typhi* (*Candida albicans* EMCC105)). The bacteria and yeast strains were cultured in nutrient broth for 24 h at 37 °C. The minimum inhibitory concentration (MIC) of reconstituted tested material against a given pathogenic strain was determined using a set of five concentrations of reconstituted tested material. One hundred microliters of the inoculums (1 × 10^8^ cfu/mL) was inculcated on agar media and poured into the Petri dish. With the help of a cork-borer (0.5 cm), a well was prepared in the plates, and 100 μL of the tested chemical was poured into it. All bacteria were cultured for 24 h at 37 °C. The diameter of the inhibition zone around the well (mm), including the well diameter, was used to calculate the zone of inhibition. The average values were tabulated after the readings were taken in triplicate in three separate fixed directions.

### 2.9. Statistical Analysis

Data were expressed as the mean ± SE. Statistical analysis was performed using one-way analysis of variance (ANOVA) and Duncan’s test as a post hoc test for the comparison of significance between groups. *p* < 0.01 was used as the statistically significant level for all analyses. For all reported mean values of measured quantities, statistically significant differences between groups are present if they are marked with different letters.

## 3. Results

To assess the effects of orally administrated KDP on mucosal immune response, we compared the mucosal IgA responses between KDP-fed mice immunized with OVA (OVA-KDP group) and positive control mice (OVA-CT group: mice administered both OVA and cholera toxin) as well as negative control mice (OVA group: mice administered OVA). Mice in the “Control” group were not immunized with OVA.

### 3.1. KDP Induced Antigen-Specific IgA Production

**Figure 2:**
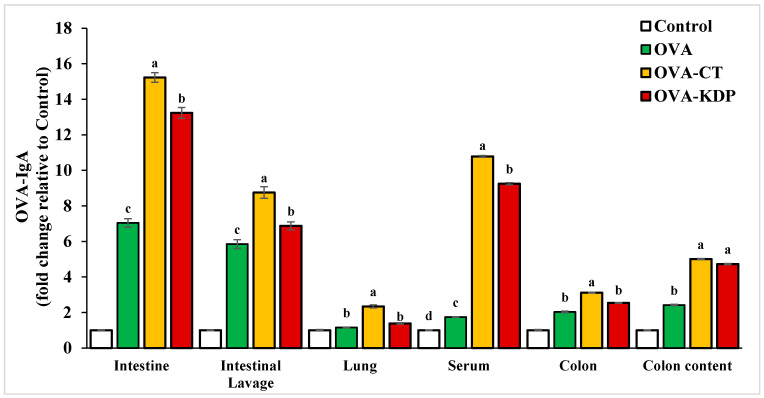
Oral administration of KDP induced OVA-specific IgA production in the small intestine, intestinal lavage, lungs, serum, colon, and colon content. Data are shown as the mean ± SE, *n* = 10. For each tissue type, mean values labeled with different letters are significantly different from each other with *p* < 0.01.

**Figure 3:**
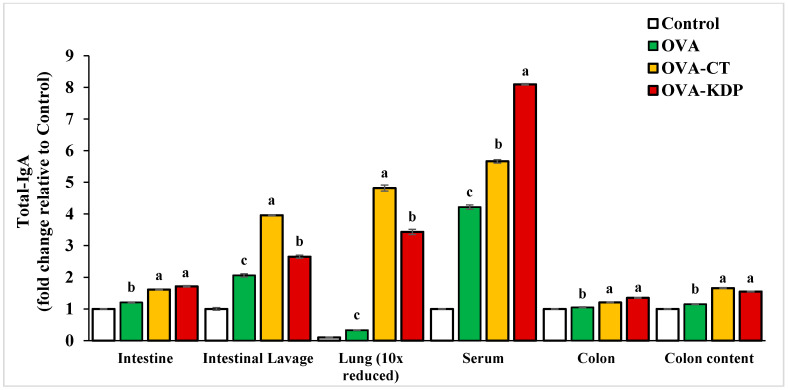
Oral administration of KDP induced Total IgA production in the small intestine, intestinal lavage, lungs, serum, colon, and colon content. Data are shown as the mean ± SE, *n* = 10. For each tissue type, mean values labeled with different letters are significantly different from each other with *p* < 0.01.

Antigen-specific IgA production was measured in each group following oral OVA immunizations. Tissue extracts of the lung, small intestine, Peyer’s patches, colon, colon content, and intestinal lavage were collected from each mouse in all groups, and then the total IgA and OVA-IgA levels were measured by ELISA. Both OVA-specific IgA and total IgA levels in the lavage fluid from small intestines, homogenized small intestines, lungs, sera, colon, and colon contents were significantly increased in the positive control group (OVA-CT) compared to the negative control (*p* < 0.01) (Figure 2 and Figure 3). Similarly, KDP oral administration exhibited a marked increase in both OVA-specific IgA and total IgA levels compared to the negative control group. It was also evident that KDP at a dose of 0.25 mg was comparable to cholera toxin in its ability to enhance the specific response in small intestines, homogenized small intestines, sera, colon, and colon contents.

### 3.2. KDP Increased Gene Expression Levels of IL-10, IL-12p40, IL-21, STAT4, and Bcl-6 in Peyer’s Patches

**Figure 4:**
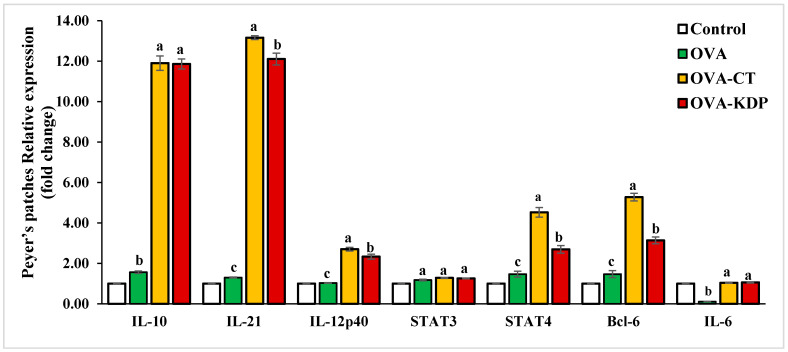
Effects of KDP on the gene expression of cytokines in Peyer’s patches cells in OVA-immunized mice. The level of gene expression was normalized to that of GAPDH mRNA expression in the control group. Data are shown as the mean ± SE, *n* = 10. For each type of expression level, mean values labeled with different letters are significantly different from each other with *p* < 0.01.

We investigated the cytokine gene expression levels in the Peyer’s patches of KDP-fed mice. Figure 4 shows that *IL-10*, *IL-12p40*, *IL-21*, *STAT4*, and *Bcl-6* gene expression were significantly increased in Peyer’s patches for the positive control group (OVA-CT) compared with expression in the negative control group; no significant change was observed in *IL-6* and *STAT3* expression. Similarly, oral administration with KDP caused a marked increase in *IL-10*, *IL-12p40*, *IL-21*, *STAT4*, and *Bcl-6* gene expression compared to the negative control, but no significant change was observed in *IL-6* and *STAT3* expression.

### 3.3. KDP Increased the Gene Expression Levels of IL-10, IL-12p40, IL-21, STAT4, and Bcl-6 in the Small Intestine

**Figure 5:**
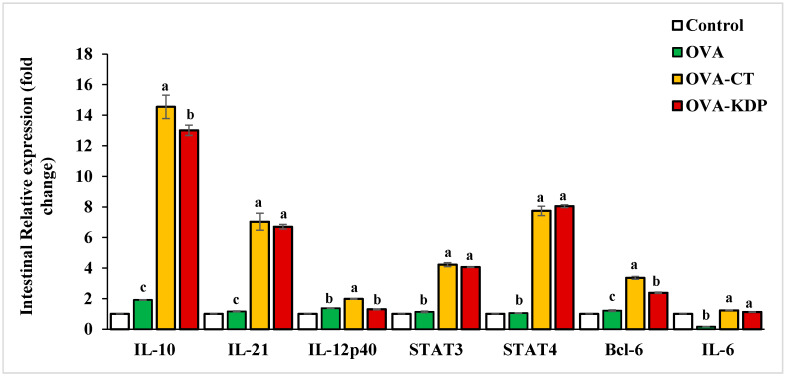
Effects of KDP on the gene expression of cytokines in small intestine cells in OVA-immunized mice. The level of gene expression was normalized to that of GAPDH mRNA expression in the control group. Data are shown as the mean ± SE, *n* = 10. For each type of expression level, mean values labeled with different letters are significantly different from each other with *p* < 0.01.

We also explored the cytokine gene expression levels in the small intestine of KDP-fed mice. Figure 5 shows that *IL-10*, *IL-21*, *STAT3*, *STAT4*, *Bcl-6*, and *IL-6* gene expression were significantly increased in the small intestine for the positive control group (OVA-CT) compared with expression in the negative control group. *IL-12p40* gene expression also increased slightly though not to statistically significant levels. Similarly, oral administration with KDP caused a marked increase in *IL-10*, *IL-21*, *STAT3*, *STAT4*, and *Bcl-6* gene expression in the small intestine compared to the negative control group.

### 3.4. KDP Enhances Antioxidant Activity

The comparison of different antioxidant assays of KDP is shown in Table 2. KDP supplementation exhibited significant inhibition by 75.56% and 72.05% for DPPH and nitric oxide, respectively. In addition, KDP inhibited a total antioxidant capacity by up to 69.01% and a reducing power by up to 66.50%. On the other hand, KDP showed lower antioxidant activities compared to standard ascorbic acid. The mean IC_50_ values of KDP are displayed in Table 3, which shows that KDP has effective radical scavenging and antioxidant activities.

### 3.5. KDP Exhibits Antimicrobial Activity

The antimicrobial activity of KDP was examined against seven samples, including three gram-negative bacteria, three gram-positive bacteria, and one yeast sample. Table 4 shows that KDP exhibited potent antimicrobial activity against all samples, though there was a difference in sensitivity among different samples toward the antimicrobial activity of KDP. The pattern of response among gram-negative bacteria was as follows: *Salmonella Typhi* > *Klebsiella pneumoniae* > *Escherichia coli*; the pattern of response among gram-positive bacteria was as follows: *Staphylococcus aureus* > *Streptococcus pyogenes* > *Bacillus cereus*. KDP exhibited a remarkable effect against *C. albicans*.

## 4. Discussion

IgA is pivotal to the intestine’s maintenance of homeostasis in the presence of microbiota. IgA is enriched in mucosal secretions, and it helps to clear and neutralize extracellular pathogens by limiting their access to the intestines and preventing their attachment to epithelial surfaces [1]. Here, ovalbumin is used to study antigen-specific immune responses because it is non-toxic and inert. Moreover, the oral administration of ovalbumin with cholera toxin or KDP to mice prevents hypo-responsiveness to OVA and induces a stronger serum IgA anti-OVA response [21,22,23]. Our results demonstrate the immunomodulatory effects by KDP as exemplified by its ability to enhance secretory IgA in mice. KDP oral co-administration with OVA exhibited a marked increase in both total IgA and OVA-specific levels compared to the negative control. The induction of total IgA production was observed in different tissues such as the small intestine, intestinal lavage, serum, lungs, colon, and colon content. These results are in accordance with recent studies demonstrating that oral intake of fermented foods, synbiotics, parabiotics, and probiotics induce a significant increase in salivary IgA secretion rate [6] and improve host defense in the gut by enhancing IgA production in the intestine [7]. Other studies have also shown that several microbial products augment mucosal immunity. *S. cerevisiae* in pig diets increases the activity of IgA and IgM against pathogens and enhances intestinal development and function [24]; *B. bifidum* increases polymeric immunoglobulin receptor expression, which in turn augments the exporting of IgA across the intestinal epithelium and increased sIgA secretion [25]; and dairy yogurt containing *B. lactis*, *L. paracasei*, and heat-killed *L. plantarum* enhances the immune function [26]. Interestingly, the use of the action of strictly defined commensal species of microorganisms that protect the intestinal mucosa by modulating the immune response may in the future become one of the forms of therapy of patients with chronic gastrointestinal diseases of various etiology, e.g., inflammatory bowel diseases [27].

The mechanism underlying KDP’s immunomodulatory effect is not known. Recent studies have shown that orally administered probiotic bacteria can interact with immune and epithelial cells in the intestine to induce cytokine and chemokine production. This interaction can in turn activate the mucosal immune system, leading to increased levels of immunoglobulin A+ cells in the intestine and other tissues [28]. Probiotics can release a variety of cytokines in a dose-dependent as well as strain-specific manner. Different cytokine responses can be induced depending on the type (gram-positive and gram-negative) and the mixture of probiotic organisms [29]. For instance, the effect of VSL#3—a special probiotic complex (consisting of four strains of Lactobacillus, three Bifidobacterium, and one strain of Streptococcus) as part of the therapy of ulcerative colitis in humans has been relatively well known. In mice, the use of VSL#3 was shown to inhibit *NF-κB* and *TNF-α* expression through the TLR4-NF-κB signaling pathway. This results in a reduction in the expression of pro-inflammatory cytokines and Toll-like receptors (TLRs) and may help to prevent exacerbations and induce remission in patients with ulcerative colitis [27]. In the current study, we examined the underlying molecular mechanisms underlying the effects of KDP on IgA production by investigating the gene expression levels of the cytokines associated with Tfh cell differentiation: *Bcl-6*, *IL-10*, *IL-12p40*, *IL-21*, and *STAT4*. Co-administration of KDP and OVA resulted in significantly increased levels in these gene expressions in the small intestine and Peyer’s patches compared to the negative control. We noted that the effect of KDP was higher in *IL-10*, *IL-21*, and *STAT4* than other cytokines. LAB can induce the production of *IL-10* and *IL-6* by dendritic cells (DCs), which stimulates sIgA production at mucosal sites in humans [30]. When *IL-10* binds to its receptor, it triggers an anti-inflammatory reaction that activates the JAK1/STAT3 pathway in which *STAT3* is phosphorylated [31]. Moreover, *IL-21* is responsible for regulating a wide variety of adaptive and innate immune responses and it has significant effects on the development of inflammatory and autoimmune disorders [32]. The role of *IL-21* in inflammatory disease of the intestine has been confirmed in studies of *IL-21*-deficient mice [33,34]. Furthermore, *IL-21* is a crucial factor in the gut mucosa response to microbiota through IgA production [35]. Earlier studies showed that *IL-21* can modulate the differentiation of B cells to IgA (+) cells by increasing the *IL-4*-driven production of IgG [36] and the TGFβ-driven production of IgA [37,38]. Finally, results of the current study show that oral co-administration of KDP and OVA increases the gene expression of B-cell lymphoma 6 (*Bcl-6*) in the small intestine and Peyer’s patches compared to the negative control. Physiologically, *Bcl-6* is a master transcription factor that can lead naïve helper T cells to differentiate into follicular helper T cells [39].

The current study also investigated KDP’s antimicrobial role against bacterial infections and its antioxidant effect. Results showed that KDP exhibits potent antimicrobial activity as evidenced by a significant decrease in the growth of seven samples of gram-negative and gram-positive bacteria and yeast, though there was a difference in sensitivity among different samples toward the antimicrobial activity of KDP. The mechanisms underlying LAB’s antibacterial activity are not fully understood but could be attributed to antioxidant capacity. Our study showed KDP supplementation exhibited a potent antioxidant activity, as indicated by significant inhibitory activity in the range of 16.52–59.39% for DPPH, nitric oxide, maximum total antioxidant capacity, and maximum reducing power. This agrees with the results of several other studies showing that LAB plays a vital role in the ameliorative antioxidant capacity in different models [40,41,42,43]. In addition, several studies have shown that different strains of LAB activate the immune system and subsequently protect the body against bacterial and viral infection [9,10,11,12,13].

Results of the current study showed that KDP enhances mucosal immunity. KDP is composed of dead cells from the bacterial strain *Lactobacillus sakei*, which are isolated from the Korean traditional food kimchi. We have recently demonstrated that the novel kefir product PFT (probiotics fermentation technology) which is a mixture that contains primarily (~90%) a heat-killed freeze-dried form of *L. kefiri* P-IF can reverse age-associated oxidative stress in mice [43] and exert an inhibitory effect against Ehrlich ascites carcinoma in mice via induction of apoptosis and immunomodulation [14]. In addition, extensive studies by others showed that heat-killed probiotics have the ability to generate beneficial biological responses [44]. These include an immunostimulatory effect in chicks by dietary supplementation of dried powder of heat-killed bacterium *E. faecalis* strain EC-12 [45] and augmentation of the non-specific immune responses in healthy dogs by supplementation of heat-killed *Enterococcus faecalis* FK-23 preparation (FK-23) [46]. In addition, the beneficial effects of non-viable probiotics on human health have been also reported [47], and the successful treatment of patients with chronic diarrhea by heat-killed *L. acidophilus* LB has been reported [48]. Furthermore, in vitro studies showed that heat-killed bifidobacteria enhances cytokine production in murine macrophage and T cell lines [49]. These studies suggest that probiotics induce their effects in both alive and heat-killed forms.

## 5. Conclusions

The results of this study indicate that KDP significantly enhances antigen-specific IgA production and gene expression in the small intestine and Peyer’s patches. In addition, KDP exhibits potent antioxidant and antimicrobial activity of several gram-negative and gram-positive bacteria and yeast. This suggests that KDP is a promising immunomodulator that can protect the intestine against pathogens.

## Figures and Tables

**Table 1:** The primer sequences.

Primer	Forward	Reverse
*IL-6* (NM_012589.2)	5′-TGGAGTCACAGAAGGAGTGGCTAAG-3′	5′-TCTGACCACAGTGAGGAATGTCAAC-3′
*IL-10* (NM_012854.2)	5′-CCAAGCCTTATCGGAAATGA-3′	5′-TTTTCACAGGGGAGAAATCG-3′
*IL-12p40* (NM_022611.1)	5′- TGGTTTGCCATCGTTTTGCTG -3′	5′-ACAGGTGAGGTTCACTGTTTCT-3′
*IL-21* (NM_001108943.2)	5′-CGCCTCCTGATTAGACTTCG-3′	5′-TGTTTCTTTCCTCCCCTCCT-3′
*IFN-γ* (NM_008337.4)	5′-CAGCAACAGCAAGGCGAAAAAGG-3′	5′-TTTCCGCTTCCTGAGGCTGGAT-3′
*STAT3* (NM_012747.2)	5′-GACCCGCCAACAAATTAAGA-3′	5′-TCGTGGTAAACTGGACACCA-3′
*STAT4* (NM_001012226.1)	5′-CATCCCTGAAAACCCTCTGA-3′	5′-GACATGGGGAGAAGGTCTGA-3′
*Bcl-6* (NM_001107084.1)	5′-CCTGAGGGAAGGCAATATCA-3′	5′-CGGCTGTTCAGGAACTCTTC-3′
*GAPDH* (NM_017008.4)	5′-TGTGTCCGTCGTGGATCTGA-3′	5′-TTGCTGTTGAAGTCGCAGGAG-3′

**Table 2:** Percent inhibition of DPPH, nitric oxide, total antioxidant capacity, and reducing power assay of KDP and ascorbic acid.

	% Inhibition							
	DPPH		Nitric Oxide		Total Antioxidant Capacity		Reducing Power Assay	
Concentration (μg/mL)	Ascorbic Acid	KDP	Ascorbic Acid	KDP	Ascorbic Acid	KDP	Ascorbic Acid	KDP
5	32.50 ^e^	11.56 ^f^	35.05 ^e^	3.50 ^f^	39.30 ^e^	11.85 ^f^	29.55 ^f^	8.00 ^f^
10	50.63 ^d^	24.06 ^e^	47.03 ^d^	15.80 ^e^	51.33 ^d^	23.03 ^e^	44.63 ^e^	17.01 ^e^
20	78.75 ^c^	31.25 ^d^	68.95 ^c^	29.09 ^d^	71.55 ^c^	35.85 ^d^	63.65 ^d^	31.90 ^d^
40	84.69 ^b^	53.13 ^c^	87.09 ^b^	42.06 ^c^	83.56 ^b^	44.79 ^c^	79.09 ^c^	43.00 ^c^
80	96.40 ^a^	66.88 ^b^	95.44 ^a^	61.94 ^b^	91.65 ^a^	56.44 ^b^	88.14 ^b^	52.04 ^b^
100	99.10 ^a^	75.56 ^a^	98.91 ^a^	72.05 ^a^	97.32 ^a^	69.01 ^a^	97.36 ^a^	66.50 ^a^

Values are expressed as the mean ± SEM, *n* = 5. Mean values followed by different superscript letters in a column are significantly different (*p* < 0.01).

**Table 3:** The IC_50_ value (μg/mL) of KDP and ascorbic acid.

	IC_50_ Value (μg/mL)			
	DPPH	Nitric Oxide	Total Antioxidant Capacity	Reducing Power
Ascorbic acid	1.74 ± 0.01 ^b^	5.98 ± 0.02 ^b^	0.11 ± 0.001 ^b^	14.70 ± 0.01 ^b^
KDP	52.90 ± 0.03 ^a^	62.46 ± 0.35 ^a^	61.15 ± 0.29 ^a^	67.92 ± 0.31 ^a^

Values are expressed as the mean ± SEM, *n* = 5. Mean values followed by different superscript letters in a column are significantly different (*p* < 0.01).

**Table 4:** Antimicrobial activity of samples of KDP.

	Sample Concentration (mg/mL)					
Pathogenic Strain	200 *	100 *	50 *	25 *	12.50 *	MIC
Gram-Negative Bacteria	Inhibition Zone Diameter (mm) **					
*Escherichia coli* ATCC25922	25 ± 0.12	23 ± 0.21	20 ± 0.23	19 ± 0.23	17 ± 0.21	12.5
*Salmonella typhi* ATCC14028	17 ± 0.03	11 ± 0.15	ND	ND	ND	100
*Klebsiella pneumoniaATCC700603*	21 ± 0.09	17 ± 0.18	13 ± 0.2	ND	ND	50
Gram-Positive Bacteria	Inhibition Zone Diameter (mm) **					
*Streptococcus pyogenes* EMCC1772	10 ± 0.05	6 ± 0.03	ND	ND	ND	100
*Staphylococcus aureus* ATCC25923	6 ± 0.02	3 ± 0.06	ND	ND	ND	100
*Bacillus cereus* ATCC10876	15 ± 0.08	13 ± 0.14	8 ± 0.17	ND	ND	50
Yeast	Inhibition Zone Diameter (mm) **					
*Candida albicans* EMCC105		7 ± 0.07	3 ± 0.03	ND	ND	50

* Concentrations of Samples and MIC are in mg/mL (*n* = 3). ** Diameter includes 5 mm well diameter. ND: Not detected. MIC: Minimum inhibition concentration.

## Data Availability

Data is available from the authors on reasonable request.
